# Draft Genome Sequence of a Methicillin-Resistant *Staphylococcus aureus* ST1413 Strain for Studying Genetic Mechanisms of Antibiotic Resistance

**DOI:** 10.1128/genomeA.00162-14

**Published:** 2014-03-06

**Authors:** Bernard S. Marasa, Javier Revollo, Saira Iram, Kidon Sung, Joshua Xu, Saeed Khan

**Affiliations:** a Division of Microbiology, National Center for Toxicological Research (NCTR), U.S. Food and Drug Administration, Jefferson, Arkansas, USA; b Division of Molecular and Genetic Toxicology, NCTR, U.S. Food and Drug Administration, Jefferson, Arkansas, USA; c Division of Bioinformatics and Biostatistics, NCTR, U.S. Food and Drug Administration, Jefferson, Arkansas, USA; d Department of Microbiology, Quaid-I-Azam University, Islamabad, Pakistan

## Abstract

Here we report the whole draft genome sequence of a methicillin-resistant *Staphylococcus aureus* ST1413 strain. Determining the distribution and arrangement of various genes associated with drug resistance, toxicity, and diseases will enhance our understanding about its adaptability to thrive in different ecological niches and help in the development of effective treatments for enterotoxigenic staphylococcal infections.

## GENOME ANNOUNCEMENT

*Staphylococcus aureus* remains one of the most important bacterial pathogens due to its high virulence and capability to cause multiple ailments ranging from complicated skin and skin structure infections to life-threatening conditions, such as endocarditis, pneumonia, and toxic shock syndrome (1, 2). *S. aureus* isolates involved in community-acquired and nosocomial infections often exhibit resistance to multiple antibiotics, including macrolides, aminoglycosides, fluoroquinolones, methicillin, and vancomycin (3–6), and pose serious challenges to treatment options. A combination of antimicrobial agents is often used to improve survival of the patients (7–9). This leads to the selection, transmission, and persistence of multidrug resistance traits in *S. aureus*. The presence of antimicrobial resistance with a wide range of enterotoxin and virulence genes makes *S. aureus* a highly pathogenic and difficult-to-treat organism. Its chromosome is reported to contain ~22% dispensable genetic material, with several regions showing large areas of genetic differences, some of which carry the genes for virulence factors, toxins, and antimicrobial resistance (10). These variations in the gene content are probably responsible for poorly understood phenomena of disease and host specificity and warrant a need to understand the genetics of *S. aureus* development and evolution. Here we report the draft genome sequence of the *S. aureus* ST1413 strain named 10S (sequence type [ST] 1413 and *spa* type t314), with approximately 2.78 million bp and 2,902 genes, 4 rRNAs, 49 tRNAs, and 1 noncoding RNA (ncRNA). *S. aureus* ST1413 lacked any detectable plasmids but was positive for staphylococcal cassette chromosome *mec* type II (SCC *mec* II), III, and V genes. It was resistant to ampicillin, tetracycline, penicillin, gentamicin, ciprofloxacin, and methicillin but sensitive to erythromycin and vancomycin and harbored toxin genes (*eta*, *sea*, *seb*, *sec*, *sei*, *sej*, *hla*, *hlb*, *hld*, *hlg*, *pvl*, *sed*, *see*, *seg*, *seh*, and *tst*), adhesin genes (*ebpS*, *bbp*, *can*, *clfA*, *clfB*, *fnbA*, *fnbB*, *map-eap*, and *spa*), and virulence genes (*ica*, *cfb*, and *v8*). Determination of the arrangement of these elements in the genome, along with their locations and genetic distributions, and comparative analyses among virulent and avirulent isolates could be extremely helpful in understanding the genetic evolution and development of effective therapeutic interventions for *S. aureus* infections.

The extraction of genomic DNA was done using a Master Pure Gram-positive DNA purification kit (Epicentre Biotechnologies). The preparation of a library, emPCR, and sequencing were performed using the GS Junior Titanium Series protocols per the manufacturer’s instructions. To prepare the library, 500 ng of genomic DNA was sheared by nebulization to obtain DNA fragments smaller than 1,000 bp. These fragments were end-repaired and ligated to Rapid Library adaptors. The library was quantitated by a fluorometer, diluted to 1 × 10^7^ molecules/μl, and used as a template for emPCR amplification. The DNA-coated beads were then primed and loaded onto a GS Junior with all the necessary sequencing reagents. A single GS Junior run generated 49,256,106 bases in 131,242 reads. The data from the run were then assembled into 327 contigs covering 2,780,250 bases by the GS De Novo Assembler version 2.7 under default settings. Annotation was performed with the NCBI Prokaryotic Genomes Automatic Annotation Pipeline (http://www.ncbi.nlm.nih.gov/genomes/static/Pipeline.html).

### Nucleotide sequence accession numbers.

This whole-genome shotgun project has been deposited at DDBJ/EMBL/GenBank under the accession number AYXU00000000. The version described in this paper is version AYXU01000000.

## References

[1] Lowy FD. 1998 *Staphylococcus aureus* infections. N. Engl. J. Med. 339:520–532. 10.1056/NEJM199808203390806 9709046

[2] Gordon RJ Lowy FD. 2008 Pathogenesis of methicillin-resistant *Staphylococcus aureus* infection. Clin. Infect. Dis. 46(Suppl 5):S350–S359. 10.1086/533591 18462090 PMC2474459

[3] de Lencastre H Oliveira D Tomasz A. 2007 Antibiotic resistant *Staphylococcus aureus*: a paradigm of adaptive power. Curr. Opin. Microbiol. 10:428–435. 10.1016/j.mib.2007.08.003 17921044 PMC2701899

[4] Rosenberg J. 1995 Methicillin-resistant *Staphylococcus aureus* (MRSA) in the community: who’s watching? Lancet 346:132–133. 10.1016/S0140-6736(95)91203-7 7603224

[5] Berger-Bächi B Rohrer S. 2002 Factors influencing methicillin resistance in staphylococci. Arch. Microbiol. 178:165–171. 10.1007/s00203-002-0436-0 12189417

[6] Mwangi MM Wu SW Zhou Y Sieradzki K de Lencastre H Richardson P Bruce D Rubin E Myers E Siggia ED Tomasz A. 2007 Tracking the *in vivo* evolution of multidrug resistance in *Staphylococcus aureus* by whole-genome sequencing. Proc. Natl. Acad. Sci. U. S. A. 104:9451–9456. 10.1073/pnas.0609839104 17517606 PMC1890515

[7] Fluit AC Wielders CL Verhoef J Schmitz FJ. 2001 Epidemiology and susceptibility of 3,051 *Staphylococcus aureus* isolates from 25 university hospitals participating in the European SENTRY study. J. Clin. Microbiol. 39:3727–3732. 10.1128/JCM.39.10.3727-3732.2001 11574603 PMC88419

[8] Eliopoulos GM. 1989 Synergism and antagonism. Infect. Dis. Clin. North Am. 3:399–406 2671129

[9] Alvarez-Lerma F. 1996 Modification of empiric antibiotic treatment in patients with pneumonia acquired in the intensive care unit. ICU-Acquired Pneumonia Study Group. Intensive Care Med. 22:387–394. 10.1007/BF01712153 8796388

[10] Fitzgerald JR Musser JM. 2001 Evolutionary genomics of pathogenic bacteria. Trends Microbiol. 9:547–553. 10.1016/S0966-842X(01)02228-4 11825715

